# Chemical organizations as a conceptual tool: from synthetic biology to interdisciplinary systems and back

**DOI:** 10.3389/fbioe.2026.1801128

**Published:** 2026-06-30

**Authors:** Tomas Veloz, Christian Jendreiko

**Affiliations:** 1 Mathematics Department, Universidad Tecnologica Metropolitana, Santiago, Chile; 2 HSD, University of Applied Sciences, Düsseldorf, Germany; 3 Centre Leo Apostel, Vrije Universiteit Brussel, Brussels, Belgium

**Keywords:** chemical organization theory, complex adaptive systems, self-organization, synthetic biology education, systems biology, pyCOT

## Abstract

This article presents a synthetic biology framework called Chemical Organization Theory (COT), that formalizes synthetic biology concepts using reaction networks. We show how this formalism applies across synthetic biology scales, from genetic circuits and metabolic engineering to synthetic consortia, and demonstrate through a dedicated computational platform (pyCOT) how abstract concepts like self-organization, emergence, feedback, and resilience become operational and computable. Drawing on 4 years of pedagogical implementation across diverse student populations—with backgrounds spanning design, social sciences, mathematics, and ecology—we find that organizational reasoning transfers effectively across disciplines. This interdisciplinary experience revealed a key pedagogical principle: students develop stronger systems thinking when they first encounter organizational concepts through familiar, non-biological systems before transferring to synthetic biology. We argue that this graduated complexity approach, enabled by COT’s domain-general formalism, addresses a fundamental gap in synthetic biology education and offers a pathway for spreading complex adaptive systems literacy beyond biology.

## 1 Introduction

### 1.1 Synthetic biology and a new education for systemic thinking

Synthetic biology has undergone a profound transformation over the past 2 decades. The field emerged with the BioBricks paradigm—assembling standardized biological parts into predictable devices (Endy, 2005). This parts-based approach, while foundational, treated biological systems as mechanical assemblies where behavior could be predicted from component specifications (Canton et al., 2008). Today’s synthetic biology tackles fundamentally different challenges: engineering minimal cells that self-replicate (Hutchison et al., 2016), designing synthetic consortia that self-organize into stable communities (Mee et al., 2014), creating evolutionary circuits that adapt to changing environments (Sleight et al., 2010), and orchestrating multi-level systems where molecular circuits shape cellular behaviors that structure ecological dynamics (Venturelli et al., 2018).

This evolution reveals a critical insight: modern synthetic biology is fundamentally about engineering *complex adaptive systems* (CAS), not merely assembling molecular components (Purnick and Weiss, 2009). Yet educational approaches remain rooted in component-level thinking. We continue teaching restriction sites, promoter sequences, and ribosome binding sites—essential knowledge—while students struggle to understand why their carefully designed circuits fail unpredictably, why metabolic pathways cannot sustain production, or why synthetic consortia collapse despite appearing well-designed (Cardinale and Arkin, 2012).

### 1.2 The educational challenge

The mismatch between synthetic biology practice and education manifests at multiple levels (Menard et al., 2024; Karim et al., 2024). We teach linear causality—signal transduction cascades drawn left-to-right, metabolic pathways as linear sequences—while synthetic biology operates through circular causality, where products regulate their own production through feedback loops (Elowitz and Leibler, 2000; Gardner et al., 2000). We teach single-level analysis—focusing on genes *or* proteins *or* cells in isolation—while synthetic biology demands multi-level reasoning, understanding how genetic circuits create cellular phenotypes that structure community dynamics (Brenner et al., 2008).

Most critically, we teach optimization of predetermined designs—finding the best parameters for a fixed circuit topology or pathway structure. Yet robust synthetic systems must exhibit self-organization and evolvability—the capacity to spontaneously form stable patterns and adapt to perturbations without explicit reprogramming (Porcar et al., 2011). This requires fundamentally different thinking: designing constraints that allow systems to organize themselves rather than specifying every detail (Levin, 2023).

The core problem is that synthetic biology practitioners need competencies that traditional molecular biology curricula do not systematically develop:

Self-organization: How do I design a system that organizes itself without external control? Metabolic flux self-balances through feedback (Stephanopoulos, 2012), synthetic consortia self-assemble into spatial patterns (Danin et al., 2010), protocells self-construct their boundaries (Luisi, 2006). Yet curricula assume designers specify everything explicitly.

Emergence: Why does not my circuit behave as the sum of its parts? Circuit behavior emerges from interaction topology and cellular context (Cardinale and Arkin, 2012), making component-level characterization insufficient for predicting system behavior (Kwok, 2010).

Multi-level selection: When engineering synthetic consortia, am I optimizing for individual cell fitness or community function? These goals often conflict (Harcombe et al., 2014), requiring explicit design to align selection pressures across scales—a competency rarely taught systematically.

Resilience: Why does my system fail when conditions change? True resilience emerges from organizational flexibility—the capacity to reorganize while preserving essential functions—rather than simple redundancy (Kitano, 2004).

This mismatch between reductionist training and complex adaptive systems practice is not unique to synthetic biology. Ecology increasingly demands network-level reasoning to understand ecosystem resilience and trophic cascades (Kauffman, 1993). Urban planning confronts self-organizing dynamics in transportation, housing markets, and community development that linear policy models fail to capture. Organizational management faces analogous challenges when designed workflows produce emergent dysfunction. The authors’ own experience confirms this. Collaborative research with ecologists modeling plant–fungus–insect networks, urban planners analyzing neighborhood revitalization dynamics, and cognitive scientists studying distributed decision-making have each revealed the same pedagogical gap—practitioners trained in component-level analysis struggling to reason about system-level viability (Veloz, 2020; Veloz and Flores, 2021). Synthetic biology is thus a particularly instructive case of a broader educational challenge: how to develop complex adaptive systems literacy across disciplines where self-organization, emergence, and multi-level dynamics are the norm rather than the exception.

### 1.3 Chemical Organization theory: a conceptual toolbox

We propose that Chemical Organization Theory (COT) can support the theoretical and pedagogical foundation that complex adaptive systems education, and particularly synthetic biology, requires. COT formalizes the autopoiesis concept—that living systems are self-producing networks (Maturana and Varela, 1980)—into a formal framework (Dittrich and di Fenizio, 2007; Veloz and Razeto-Barry, 2017). The core insight is elegant: systems are networks of reactions transforming resources; stable patterns are *organizations*—subsets that are simultaneously closed (producing no new resource types) and self-maintaining (regenerating everything they consume).

This formalism captures the essence of what synthetic biologists actually do: engineer self-maintaining networks that exhibit closure under operational conditions. A genetic circuit is an organization of genes, proteins, and regulatory interactions. A metabolic pathway is an organization of enzymatic reactions. A minimal cell is an organization encompassing metabolism, information processing, and boundary formation. A synthetic consortium is an organization of organizations—multiple cellular systems maintaining each other through exchange.

COT provides synthetic biology with a formal language that applies from molecular circuits to ecological communities, makes abstract concepts like self-organization and emergence operational and computable, and guides design by revealing which network structures can form stable organizations. In this way, COT identifies structurally viable designs before investing in construction and kinetic optimization. Unlike existing pedagogical frameworks that describe synthetic biology across scales (Karim et al., 2024) or survey evolving teaching practices (Menard et al., 2024), COT provides a *formal, computable* framework that makes organizational reasoning precise and actionable.

### 1.4 Structure and contributions

This paper makes three contributions to synthetic biology education. First, we establish that synthetic biology at every scale is fundamentally organizational engineering (Section 2). Second, we present the core formalism of COT and show how it operationalizes some essential concepts for synthetic biology practice (Sections 3, 4). Third, we illustrate through concrete examples how COT applies to genetic circuits, metabolic engineering, minimal cells, and synthetic consortia, and discuss pedagogical experiences implementing COT-based education (Sections 5, 6).

## 2 Complex adaptive systems and self-organization in synthetic biology

### 2.1 Fundamental characteristics of engineered living systems

Synthetic biology systems, whether genetic circuits or synthetic ecosystems, exhibit properties that distinguish them from traditional engineered systems (Heinemann and Panke, 2006). Understanding these properties is essential for effective design.

#### 2.1.1 Contextual dynamics

Small changes in cellular context or environmental conditions can produce disproportionately large effects on circuit behavior, while large perturbations may have minimal impact (Cardinale and Arkin, 2012). This non-linearity challenges the engineering assumption that systems respond proportionally to inputs. For example, a promoter’s behavior can change dramatically when moved between genomic locations or when host cell state shifts, despite identical molecular components (Canton et al., 2008).

#### 2.1.2 Feedback loops

Positive feedback amplifies changes, potentially leading to bistability or oscillations, while negative feedback provides stabilization through homeostatic mechanisms (Gardner et al., 2000). The interplay between feedback types creates the dynamic balance between stability and change that characterizes living systems. Synthetic circuits explicitly leverage feedback: toggle switches use mutual repression (positive feedback) for bistability (Gardner et al., 2000), repressilators use cyclic repression for oscillation (Elowitz and Leibler, 2000), and metabolic pathways employ feedback inhibition for flux control (Stephanopoulos, 2012).

#### 2.1.3 Self-organization

The ability to spontaneously form stable patterns without external control or predetermined blueprints (Ashby, 2017). In synthetic biology, this manifests as metabolic networks spontaneously balancing flux through feedback (Stephanopoulos, 2012), cells in consortia spontaneously organizing into spatial patterns (Danin et al., 2010), and minimal cells spontaneously forming boundaries that define their identity (Luisi, 2006).

#### 2.1.4 Emergent properties

System-level behaviors that cannot be predicted from knowledge of individual components alone (Heyli and ghen, 2023). These properties are well studied in the nonlinear dynamics literature (Strogatz, 2000; Kauffman, 1993; Tyson et al., 2003), which provides the mathematical foundations for understanding how feedback, bifurcations, and attractors shape parts-to-collective behavior.

Table 1 summarizes these fundamental characteristics and their implications for synthetic biology design.

**TABLE 1:** Features of synthetic biology systems.

Feature	Ex. In SynBio	Design challenge
Contextuality	History-dependence	Predictability
Feedback	Homeostasis	Feedback strength
Self-organization	Feedback co-regulation	Collective constraints
Emergence	Top-down control	Multi-scale modeling

### 2.2 Self-organization as a design principle

Self-organization represents a paradigm shift from traditional engineering (Von Foerster, 2003). Rather than specifying every system detail, designers create conditions that allow desired patterns to emerge spontaneously. Certain interesting features of this way of thinking:

- **Biological complexity exceeds design capacity**: Even simple cells involve thousands of interacting components. Specifying all interactions explicitly is impossible. Self-organizing designs leverage cellular machinery to handle complexity (Porcar et al., 2011).
- **Evolution requires flexibility**: Fixed designs fail when conditions change. Self-organizing systems adapt by transitioning between stable states (Sleight et al., 2010). Evolutionary robustness comes from organizational flexibility, not rigid optimization.
- **Scale-up demands autonomy**: Large-scale bioprocessing cannot rely on continuous external control. Systems must self-regulate (Stephanopoulos, 2012).

The process of self-organization in synthetic systems typically involves: (1) initial perturbation creating instability (circuit induction, metabolite accumulation, consortium assembly), (2) amplification through positive feedback selecting certain configurations over others, and (3) stabilization through negative feedback locking in successful patterns (Ashby, 2017). Understanding this process allows designers to guide self-organization toward desired organizational states.

### 2.3 The central question: what makes systems viable?

The fundamental question in synthetic biology is not ”how do I build this component?” but ”will this network of interactions form a stable, functional system?” (Kwok, 2010). A genetic circuit is viable if it reaches stable expression states. A metabolic pathway is viable if it sustains flux without depleting essential intermediates. A minimal cell is viable if it maintains metabolism, replication, and boundary integrity. A synthetic consortium is viable if all member species persist through balanced exchange.

Traditional approaches answer this question through detailed simulation—solving coupled differential equations describing every molecular interaction (Strogatz, 2000). This becomes computationally intractable for complex systems and requires precise kinetic parameters rarely available (Heinemann and Panke, 2006). COT offers an alternative: analyze network structure to determine which configurations are *organizationally viable*—capable of forming closed, self-maintaining patterns—regardless of detailed kinetics. This qualitative analysis is essential during a design phase, before investing in construction and characterization which can require dealing with multiple and complicated details.

## 3 Reaction networks and chemical organizations

### 3.1 Reaction networks: basic formalism

A reaction network consists of two fundamental elements: *resources* and *reactions*. Resources correspond to molecular species in the original COT format, but in general can be thought as of any kind of entity subjected to transformation, while reactions correspond to such elementary processes that transform resources (Veloz and Razeto-Barry, 2017).

Formally, a reaction network is defined as ⟨M,R⟩ , where $M=\left\{s_{1},s_{2},\ldots \right\}$ is the set of resources, and let $N^{M}$ denote the set of multisets over M , i.e., functions $M\to N_{\geq 0}$ assigning a non-negative integer multiplicity to each resource. The set of reactions $R\subseteq N^{M}\times N^{M}$ consists of pairs of multisets. Each reaction r∈R maps a multiset of resources into another multiset:

$$r:a_{1}s_{1}+a_{2}s_{2}+\cdots \to b_{1}s_{1}+b_{2}s_{2}+\cdots$$

where $a_{i},b_{i}\in N_{\geq 0}$ are stoichiometric coefficients specifying how many units of each resource are consumed and produced, the ’+’ symbol represents conjunction (all inputs must be simultaneously present), → denotes the transformation process. The use of multisets rather than sets is essential: stoichiometric coefficients distinguish reactions like $2s_{1}\to s_{2}$ from $s_{1}\to s_{2}$ , a distinction critical for self-maintenance analysis.

In synthetic biology contexts, resources can represent:

- **Genetic circuits**: DNA, mRNA, proteins, transcription factors, regulatory states
- **Metabolic engineering**: substrates, intermediates, products, cofactors, enzymes
- **Minimal cells**: metabolites, information molecules (DNA/RNA), membrane components
- **Synthetic consortia**: cell types, extracellular metabolites, signaling molecules
- **Ecological systems**: ecological species, resources, environmental conditions

### 3.2 Organizations as design targets

The central concept in COT is an *organization*—a subset of resources and reactions that is simultaneously *closed* and *self-maintaining* (Dittrich and di Fenizio, 2007). For synthetic biologists, organizations represent the stable configurations their designs can achieve.

To specify these definitions precisely:

- Let X⊆M be a subset of resources (species)
- The set of reactions that can be triggered from species in X is denoted $R_{X}$

- Species consumed by reactions in $R_{X}$ : $\text{supp}\left(R_{X}\right)$

- Species produced by reactions in $R_{X}$ : $\text{prod}\left(R_{X}\right)$

-
X is closed if and only if $\text{prod}\left(R_{X}\right)\subseteq X$ (no novel species are produced)
-
X is semi-self-maintaining if and only if $\text{supp}\left(R_{X}\right)\subseteq \text{prod}\left(R_{X}\right)$ (qualitatively, everything consumed is also produced)
-
X is self-maintaining if there exists a positive flux vector $\text{v}\in R_{>0}^{\left|R_{X}\right|}$ over the reactions in $R_{X}$ such that S⋅v≥0 for all species in X , where S is the stoichiometric matrix restricted to the species in X and the reactions in $R_{X}$ . In other words, production rates can quantitatively compensate consumption rates under some sustainable flux regime which ensures all reactions that can possibly occur do occur at a positive rate. This condition is decidable *via* linear programming (Dittrich and di Fenizio, 2007)
-
X is an Organization if and only if it is both Closed and Self-maintaining

#### 3.2.1 A critical distinction for synthetic biology design

Semi-self-maintenance (qualitative cycles exist) does not guarantee self-maintenance (quantitative sustainability). Consider a toy metabolic network with $M=\left\{s_{1},s_{2}\right\}$ and reactions $r_{1}:s_{1}\to s_{2}$ and $r_{2}:2s_{2}\to s_{1}$ . The full set M is closed and semi-self-maintaining ( $s_{1}$ consumed by $r_{1}$ is produced by $r_{2}$ ; $s_{2}$ consumed by $r_{2}$ is produced by $r_{1}$ ). However, M is not self-maintaining because every cycle loses one $s_{2}$ molecule—quantitative production cannot balance consumption. To see this formally, the stoichiometric matrix is $\text{S}=\left(\begin{array}{cc} -1 & 1\\ 1 & -2 \end{array}\right)$ for species $\left(s_{1},s_{2}\right)$ and reactions $\left(r_{1},r_{2}\right)$ . For any non-negative flux vector $\text{v}={\left(v_{1},v_{2}\right)}^{T}$ , the net production of $s_{2}$ is $v_{1}-2v_{2}$ . Since $r_{2}$ requires two units of $s_{2}$ but $r_{1}$ produces only one, no positive flux through both reactions can achieve S⋅v≥0 .

A complementary example clarifies the relationship between organizational viability and kinetic realizability. Consider $M=\left\{s_{1},s_{2}\right\}$ with reactions $r_{1}:s_{1}\to s_{2}$ and $r_{2}:s_{2}\to s_{1}$ . The stoichiometric matrix is $\text{S}=\left(\begin{array}{cc} -1 & 1\\ 1 & -1 \end{array}\right)$ . Note that $s_{1}$ and $s_{2}$ can self-maintain in this case as the cyclic pathway $\left(r_{1},r_{2}\right)$ has zero-net change. Formally, for any process $\text{v}={\left(v,v\right)}^{T}$ with any v>0 we obtain S⋅v=0 , satisfying the self-maintenance condition. The set $M=\left\{s_{1},s_{2}\right\}$ is therefore an organization. However, suppose the reaction dynamics is such that with $v_{i}=k_{i}\cdot s_{i}$ for i=1,2 . This dynamical model is known as the mass-action kinetics and it is the most common way in which reaction networks dynamics is modeled (Feinberg, 2019). The self-maintaining condition requires $v_{1}=v_{2}$ , i.e., $k_{1}\cdot s_{1}=k_{2}\cdot s_{2}$ . However, if our system requires $k_{1}>k_{2}$ and $s_{1}>s_{2}$ (for example, to maintain a concentration gradient favoring forward process), then $k_{1}s_{1}>k_{2}s_{2}$ implying the self-maintaining process cannot be dynamically realized. This illustrates a key lesson: COT certifies that a *structurally feasible* process exists, but whether specific kinetic parameters and concentration constraints can instantiate that regime is a separate question requiring dynamical analysis. Organizational viability is necessary but not sufficient for a functioning system under arbitrary kinetic specifications.

This teaches designers a crucial lesson: *not all feedback loops are sustainable*. Many failed metabolic engineering attempts result from designs that appear cyclical (semi-self-maintaining) but cannot sustain flux (not self-maintaining) due to structural imbalances or thermodynamic constraints rendering self-maintaining processes infeasible (Stephanopoulos, 2012).

### 3.3 Advantages for synthetic biology modeling

Reaction networks provide a clearer description than traditional network representations (Heylighen et al., 2024). When multiple regulatory edges converge on a gene, do they represent logical AND (all required) or OR (any sufficient)? Reaction networks resolve this explicitly: ’+’ within reactions is AND, multiple reactions producing the same output represent OR.

Consider designing a synthetic AND gate where output protein y is produced only when both inputs x and u are present:

- Correct specification: x+u→y (both required)
- Incorrect specification would be: x→y ; u→y (either suffices)

Beyond clarifying the transformative role of reactions, COT brings further advantages:

#### 3.3.1 Qualitative analysis without kinetic parameters

Most synthetic biology designs lack precise kinetic parameters during the design phase (Canton et al., 2008). COT enables structural analysis—identifying which network topologies are viable—without requiring parameter values (Dittrich and di Fenizio, 2007). This is exactly what designers need: knowing which designs can work before investing in construction.

#### 3.3.2 Scalability

Algorithms efficiently compute organizations for networks with hundreds to thousands of species (Centler et al., 2008; Peter et al., 2023). This enables realistic modeling of metabolic networks, gene regulatory cascades, and multi-species consortia—systems too complex for manual analysis.

#### 3.3.3 Multi-scale integration

The same formalism applies from molecular circuits to ecological communities (Veloz and Razeto-Barry, 2017; Peter et al., 2023). A cell is an organization of molecular reactions; a consortium is an organization of cellular organizations. This enables coherent multi-scale design.

#### 3.3.4 Design-oriented analysis

**FIGURE 1:**
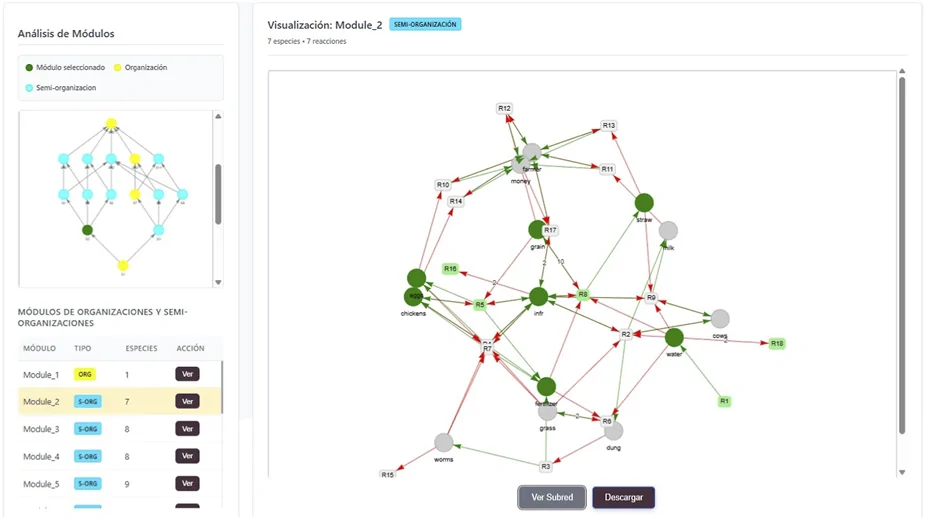
Hierarchical analysis based on pyCOT web application of a reaction network model of a Farm developed in (Veloz et al., 2022): Left-top shows the hierarchy of organizations, the bottom list enables user to pick one organization (marked in green in the hierarchy). The right shows the part of the reaction network forming the organization in green nodes, and gray nodes represent the species that are absent in the organization, enabling to discern the organization from the full network.

COT identifies the organizational structure of a reaction network: the set of organizations and their hierarchical containment relations. Each organization corresponds to a region of state space where the active species can persist indefinitely under some flux regime—analogous to fixed points, limit cycles, or invariant sets in ODE models (Strogatz, 2000). An organization is hence a potential persistent state—a structural prerequisite for a stable attractor. Since smaller organizations can be contained in larger ones, this structure forms a hierarchy of potential long-term behaviors. Importantly, COT identifies this hierarchy without requiring kinetic parameters, though the time-scales and basins of attraction associated with each organization require kinetic analysis. This directly addresses the synthetic biologist’s question: “could my design work?” rather than “exactly how will concentrations change over time?” In fact, whether an organization acts as an attractor (drawing nearby trajectories toward it) is a kinetic-specific question that requires further analysis (Peter and Dittrich, 2011). Figure 1 illustrates this organizational hierarchy as computed by the pyCOT platform. This visual representation makes the abstract notion of organizational structure immediately tangible for students and designers alike.

### 3.4 Why organizations capture synthetic biology’s essence

Organizations formalize the properties that make synthetic biology systems functional:

#### 3.4.1 Closure defines the possibility space

In a genetic circuit, closure means the circuit produces only the intended components—no unexpected protein products, no toxic intermediates. In a metabolic pathway, closure means no unwanted side products accumulate. In a minimal cell, closure means all essential molecules can be produced from available building blocks. Violations of closure cause design failures: unexpected products, toxic accumulation, or essential components that cannot be synthesized.

#### 3.4.2 Self-maintenance defines sustainability

In a genetic circuit, self-maintenance means the circuit can regenerate its components despite degradation—proteins are continuously produced to balance proteolysis. In a metabolic pathway, self-maintenance means cofactors like ATP and NADH are regenerated—the pathway does not deplete the cell’s energy currency. In a synthetic consortium, self-maintenance means each species’ growth is sustained by metabolites from others—no species starves or overgrows. Violations of self-maintenance cause pathway shutdown, cellular dysfunction, or consortium collapse.

#### 3.4.3 Organizational hierarchy captures multi-level structure

Small organizations (e.g., individual metabolic modules) nest within larger ones (complete metabolism), which nest within still larger ones (cell, then consortium). This hierarchy reveals natural decomposition into functional subsystems and enables modular design (Veloz and Razeto-Barry, 2017).

### 3.5 Resilience through organizational dynamics

COT provides sophisticated framework for understanding resilience in synthetic biology (Veloz et al., 2023; Heylighen et al., 2022):

#### 3.5.1 Local resilience

An organization’s immediate capacity to return to its original state after perturbation. Genetic circuits with high local resilience have multiple redundant pathways to restore stable states. Metabolic networks with high local resilience can compensate for enzyme knockouts through alternative routes.

#### 3.5.2 Global resilience

An organization’s long-term viability within the evolutionary process. Even locally unstable organizations may have high global resilience if they represent frequently visited configurations. This is crucial for evolutionary robustness (Sleight et al., 2010).

#### 3.5.3 Adaptive regulation

Organizations demonstrate cybernetic properties through nested feedback loops. When perturbations reduce critical resources, reaction rates automatically adjust, creating homeostatic responses that emerge from network structure (Ashby, 2017).

#### 3.5.4 Evolvability

The capacity for ”minimal reorganization”—transitioning to related organizational forms while preserving core functionality (Heylighen et al., 2024). Highly evolvable networks have rich organizational landscapes with multiple overlapping organizations, enabling adaptation while maintaining viability.

#### 3.5.5 Distributed organization

Multiple closed sets that collectively self-maintain without any single set being fully self-maintaining (Peter et al., 2023). This concept challenges traditional notions of individuality and is particularly relevant for synthetic consortia where collective function does not require individual autonomy.

## 4 Applications across synthetic biology scales

### 4.1 Genetic circuits: organizations as stable states

The toggle switch (Gardner et al., 2000) demonstrates organizational thinking in circuit design. Rather than viewing it as ”mutual repression creates bistability,” we recognize that *two organizations coexist*:

Consider resources $M=\left\{RNAP,gene_{1}^{\mathrm{free}},gene_{1}^{\mathrm{blocked}},gene_{2}^{\mathrm{free}},gene_{2}^{\mathrm{blocked}},rep_{1},rep_{2}\right\}$ where $gene_{i}^{\mathrm{free}}$ represents gene i with free (unblocked) promoter, and $gene_{i}^{\mathrm{blocked}}$ represents gene i with promoter bound by repressor. Reactions:

$$gene_{1}^{\mathrm{free}}+RNAP\to gene_{1}^{\mathrm{free}}+RNAP+rep_{1}$$

$$gene_{2}^{\mathrm{free}}+RNAP\to gene_{2}^{\mathrm{free}}+RNAP+rep_{2}$$

$$gene_{2}^{\mathrm{free}}+rep_{1}\to gene_{2}^{\mathrm{blocked}}+rep_{1}$$

$$gene_{1}^{\mathrm{free}}+rep_{2}\to gene_{1}^{\mathrm{blocked}}+rep_{2}$$

$$rep_{1}\to \emptyset \left(\text{degradation}\right)$$

$$rep_{2}\to \emptyset \left(\text{degradation}\right)$$

Two organizations exist:

-
$O_{1}=\left\{RNAP,gene_{1}^{\mathrm{free}},gene_{2}^{\mathrm{blocked}},rep_{1}\right\}$ : Gene 1 ON, produces $rep_{1}$ which keeps gene two blocked
-
$O_{2}=\left\{RNAP,gene_{2}^{\mathrm{free}},gene_{1}^{\mathrm{blocked}},rep_{2}\right\}$ : Gene 2 ON, produces $rep_{2}$ which keeps gene 1 blocked

The insight: *Bistability requires the coexistence of two competing organizations*. Any network with two non-overlapping organizations is a *candidate* for exhibiting bistability—having two organizations is a necessary structural condition. Whether these organizations correspond to dynamically stable steady states depends on kinetic properties (reaction rates, degradation constants) not captured by organizational analysis alone. Nevertheless, COT transforms circuit design from template-following to principle-based reasoning: to create bistability, first ensure the network topology supports at least two non-overlapping organizations, then analyze stability within each. Networks lacking this structural prerequisite cannot exhibit bistability regardless of parameter tuning.

The repressilator (Elowitz and Leibler, 2000) demonstrates a complementary insight: sustained oscillation requires that *no stable organization exists*. The cyclic repression network is designed so closure cannot be achieved—every configuration produces species preventing its own maintenance, forcing perpetual transitions. While the repressilator’s specific oscillatory dynamics depend on parameter regimes, COT identifies the *absence of stable steady states*—a necessary condition for oscillation. Other dynamical behaviors such as chaotic dynamics or divergence are also structurally possible when no stable organization exists; the specific outcome depends on the kinetics.

### 4.2 Metabolic engineering: organizational viability

Consider engineering *E. coli* for biofuel production *via* fatty acid synthesis pathway (Wakil, 1961). Traditional approach: clone pathway genes, optimize expression, iterate based on titers. Why do designs fail unpredictably?

Organizational analysis reveals failure modes:

**Failure Mode 1: Closure violation**—Pathway produces intermediate X that native metabolism cannot process, X accumulates toxically.

**Failure Mode 2: Self-maintenance violation**—Pathway consumes NADPH faster than native metabolism regenerates it, cofactor depletion shuts down pathway.

**Successful design criteria**: Target product must appear in an organization. This requires:

1. Closure: All pathway intermediates either convert to product or are metabolized by native pathways (no toxic accumulation)
2. Self-maintenance: Cofactor regeneration balances consumption (ATP, NADH, NADPH cycles close)
3. Integration: Heterologous pathway forms organization with native metabolism (compatible flux)

This enables *predictive design*: compute organizations before construction. If target product does not appear in any organization, the design will fail. Iterate computationally, not experimentally.

### 4.3 Minimal cells: finding minimal organizations

What is the minimal metabolic network capable of sustaining life? (Hutchison et al., 2016). COT reframes this as: what is the *smallest organization* encompassing essential functions (energy, building blocks, information, boundaries)?

#### 4.3.1 Design challenge

Given available reactions, identify minimal subsets forming organizations that include {ATP, amino acids, nucleotides, lipids}.

Many proposed minimal cell designs fail this test: they produce unsustainable components (closure violations) or require external resources that cannot be regenerated (self-maintenance violations). COT provides the formal criterion for viability: the network must form an organization.

To illustrate COT’s applicability beyond molecular-scale networks, consider the farm model developed in (Veloz et al., 2022). In this reaction network, resources include grass, water, cows, chickens, grains, milk, and eggs, while reactions represent productive processes such as feeding, milking, egg collection, crop cultivation, and resource replenishment. Organizational analysis identifies which subsets of the farm system are self-maintaining—for example, a milk-producing organization requires that grass regeneration and water availability sustain cow feeding at rates that balance consumption, forming a closed productive cycle. The study further demonstrates how three fundamental types of perturbation—state (e.g., reduction in water quantity), process (e.g., climate change constraining water input below a critical threshold), and structural (e.g., loss of a reaction due to disease eliminating a species)—affect the farm’s organizational viability. By decomposing the network into dynamically decoupled modules, the analysis reveals which perturbations can be compensated within the existing organizational structure and which force transitions to alternative, possibly diminished, organizations. This example demonstrates that COT scales naturally from molecular networks to socio-ecological systems, reinforcing its utility as a general framework for analyzing viability and resilience across scales—a property that proves particularly valuable in pedagogical settings where students encounter COT first through non-biological systems (Section 5).

### 4.4 Synthetic consortia: multi-level organizations

Engineering stable multi-species systems (Venturelli et al., 2018) requires organizational thinking at two levels:

Individual level: Each species is a molecular organization (metabolism, regulation, structure).

Community level: The consortium is an ecological organization (collection of species plus exchange reactions).

Consider a three-species degradation consortium: Species A degrades complex substrate to intermediate, B degrades intermediate to byproduct, C mineralizes byproduct. Why do such consortia often fail (Harcombe et al., 2014)?

Organizational failure modes:

1. Missing closure: Species A produces metabolite X unused by B or C, X accumulates toxically
2. Imbalanced self-maintenance: Species C grows faster than A supplies substrate, C starves, consortium collapses
3. Fragile organization: Perturbation shifts to alternative organization where B dominates, outcompeting others

Ensure community-level organization aligns with individual fitness:

- Obligate mutualisms: Make individual viability dependent on community (neither species self-maintains alone)
- Spatial structure: Local interactions favor community organizations
- Distributed organization: Collective self-maintenance without individual autonomy (Peter et al., 2023)

## 5 Pedagogical applications: teaching synthetic biology through organizations

### 5.1 Implementation across institutions

Since May 2021, we have implemented COT-based synthetic biology education across three institutions with diverse student populations:

#### 5.1.1 HSD (Düsseldorf)

Semester courses in Communication Design and the MA program Transforming Digitality taught by Prof. Jendreiko. Students had no prior biology training; backgrounds included graphic design, communication studies, social sciences, and engineering. 15–20 students per cohort. Regular offering since 2021.

#### 5.1.2 VUB (Brussels)

LEAP research group within Centre Leo Apostel directed by Prof. Veloz. 10+ Masters and PhD students from diverse areas including urban planning, statistics, cognitive science, computational biology, data science, philosophy and interdisciplinary studies—with high variance on formal biology training as some students have strong training and others none. Bi-weekly meetings since 2021, specialized seminars and two workshops a year (on average) to discuss COT related work.

#### 5.1.3 UTEM (Santiago)

MA Mathematical Biology program where Prof. Veloz teaches. Students had mathematics backgrounds with introductory biology exposure. Thesis advising and specialized seminars. Six students (2 graduated, 4 in progress).

Notably, our formal pedagogical cohorts did not include students with exclusive prior training in molecular biology or synthetic biology, but mostly mixed cohorts. However, the authors have applied reaction network and COT-based reasoning in research collaborations with ecologists, developmental biologists, epidemiologists, and mathematical biologists (Veloz, 2020; Veloz and Flores, 2021). In each case, organizational thinking provided an effective bridge between disciplinary expertise and systems-level reasoning, suggesting that the framework facilitates systemic design regardless of background—though we emphasize that this evidence is anecdotal and drawn from research rather than controlled pedagogical settings. Systematic investigation of how COT-based instruction interacts with established reductionist training in biology students is an important direction for future work. Similarly, our current curriculum does not yet systematically teach complementary methods for integrating COT with dynamical methods such as stochastic modeling. Integrating such follow-on modules is a natural next step.

### 5.2 Facilitating a graduated complexity pedagogical framework

Our central finding: Progressive development of organizational intuition requires starting with familiar domains, then formalization in simple cases to gain confidence with COT principles. Only in a third stage it is possible to achieve effective transfer to specific phenomena, either biological or otherwise. We prefer then to divide the pedagogical approach in three stages:

#### 5.2.1 Stage 1: discovering complex adaptive systems features through familiar systems

Students model systems they know intimately from their professional and everyday domains. The goal is not yet formal modeling but *recognition*—discovering that feedback, self-organization, closure, and emergence already operate in systems they understand intuitively. Across our three cohorts, this stage produced remarkably diverse entry points:

- **HSD (Düsseldorf)**: Students of Communication Design as well as students in the interdisciplinary Master’s Program Transforming Digitality, from diverse backgrounds including design, architecture, engineering, and social sciences, modeled systems of diverse nature. A first group modeled dance choreographies as reaction networks, where body positions are resources and movement transitions are reactions—revealing that sustained dance phrases require organizational closure (every position must be reachable from others). A second group modeled facial expressions of emotions, identifying how combinations of facial cues produce and reinforce emotional displays on others, forming feedback loops. A third group modeled Minecraft farming mechanics, discovering that sustainable virtual farms require self-maintaining cycles of planting, harvesting, composting, and seed recovery—a direct analogy to metabolic self-maintenance.
- **VUB (Brussels)**: Two experiences are remarkable: A PhD student in data science developed an interdisciplinary climate change model, mapping carbon sources, sinks, policy interventions, and socioeconomic feedbacks as a reaction network whose organizational structure revealed which mitigation strategies were self-sustaining *versus* dependent on continuous external input. A postdoctoral student introduced similar ideas to model physical and psycological factors combined to reproduce conflict dynamics, leading to a collaboration with the Norwegian Institute of International Affairs.
- **UTEM (Santiago)**: Students from the Masters in biomathematics received training for modeling ecological systems at multiple scales. One project modeled a micro-ecosystem of plants, mycorrhizal fungi, soil bacteria, and pollinating insects, where organizational analysis identified which species subsets could persist without external supplementation. A larger-scale project modeled a riparian ecosystem involving beavers, native trees, water wells, *diques* (beaver dams), and bird species, showing how beaver dam-building creates new organizations by generating water retention resources that sustain plant and bird communities—but also collapse insect and birds populations whose self-maintenance depends on trees beavers use to create dams.

In every case, the key cognitive shift occurred when students discovered that a variety of circular causality is the deep cause of systems persistence, and not the traditional sense of stability as ’push-back’ proportional to perturbation. Dance students realized how dance initial conditions set different choreographic feedback loops; Minecraft farmers discovered that soil depletion justify multiple alternatives such as nomad activities, land occupation, recuperation techniques, *etc.*

#### 5.2.2 Stage 2: formalizing organizational principles through COT

While exploring familiar systems, students simultaneously learn the formal apparatus of COT using pyCOT (pycot.utem.cl, see Figure 1). Interactive visualization of organizational hierarchies makes abstract concepts tangible. Students explore perturbation simulations showing system reorganization, and complete design challenges such as “create a network with exactly 3 organizations in a specific hierarchy.” The parallel timing is deliberate: students continuously translate between their intuitive familiar-system models and the formal COT representations, strengthening both.

#### 5.2.3 Stage 3: transfer to specific phenomena

With intuition and formalism established, students model systems of increasing biological complexity: genetic circuits (toggle switches, repressilators), metabolic pathways (cofactor balancing, pathway integration), minimal cell architectures, and synthetic consortia. Crucially, transfer is not restricted to biology—VUB students have applied organizational analysis to urban infrastructure networks and creative production workflows, while UTEM students have modeled ecological management interventions. Assessment at this stage focuses on whether students can identify organizations without guidance and spontaneously apply organizational reasoning to predict design failures in unfamiliar systems.

## 6 Discussion and future directions

### 6.1 A good basis for synthetic biology

We propose that synthetic biology, across all its diverse manifestations, can be benefited from the complex adaptive systems principles. The latter is expressed as the engineering of self-maintaining, organizationally closed reaction networks.

Parts-based synthetic biology provides essential vocabulary—resources and transformations—but vocabulary alone cannot generate viable systems. Well-characterized components must be composed into networks satisfying organizational constraints: closure and self-maintenance. One source of the recurring frustration—that characterized parts fail when combined—is the gap between having individual functional components and having organizations. Parts-based designs can fail for many reasons—context dependence (Cardinale and Arkin, 2012), measurement uncertainty, evolutionary instability (Sleight et al., 2010), metabolic burden, and others. Organizational incoherence—the absence of closure or self-maintenance in the combined network—represents one specific and structurally diagnosable category of failure that COT addresses.

Whole-genome engineering exemplifies this directly. The JCVI-syn3.0 minimal genome (Hutchison et al., 2016) asks: what is the smallest gene set whose products form a self-maintaining, closed network? COT transforms ”what genes do we need?” into ”what is the minimal organization supporting life?” enabling computational search before expensive synthesis.

Cell-free synthetic biology explores organizational principles where cellular complexity does not obscure fundamentals (Silverman et al., 2019). By reconstituting networks *in vitro*, researchers test whether designs achieve predicted organizational properties.

Synthetic ecology represents explicitly multi-level organizational engineering (Venturelli et al., 2018). Each species is an organization; the community forms a higher-level organization. The design challenge is aligning organizations across scales. When alignment fails, consortia collapse.

### 6.2 Critical reflection: what works and what doesn’t

**FIGURE 2:**
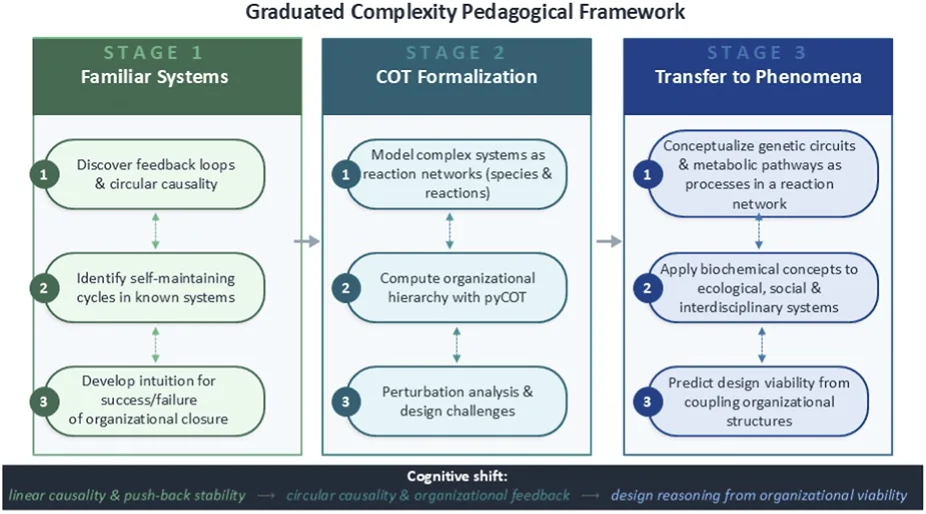
Graduated complexity framework for COT-based education. Students first develop organizational intuition by modeling familiar non-biological systems (Stage 1), then formalize this intuition using reaction networks and the pyCOT platform (Stage 2), and finally transfer organizational reasoning to synthetic biology and interdisciplinary applications (Stage 3). The framework produces a cognitive shift from linear causality toward design reasoning grounded in organizational viability.

Four years of implementation reveal consistent patterns in what facilitates and what hinders student learning. Graduated complexity—beginning with familiar systems before biological applications—proves essential rather than optional, as direct biological teaching overwhelms students with dual cognitive loads. Peer learning through self-organizing groups accelerates acquisition, while interactive visualization tools make abstract organizational concepts tangible. However, challenges persist: mathematical anxiety affects some students. In these cases is where tools such as pyCOT and the manual constuction of reaction networks using objects proves especially useful. Table 2 summarizes key pedagogical lessons, complementing the visual overview of the graduated complexity framework in Figure 2.

**TABLE 2:** Key pedagogical lessons from COT-Based synthetic biology education.

Successful strategies	Persistent challenges
Graduated complexity: Start with familiar systems, not biology	Mathematical anxiety despite avoiding differential equations
Peer learning: Self-organizing groups accelerate acquisition	Time investment: 8–10 weeks minimum for scaffolding
Interactive visualization: pyCOT makes concepts tangible	Transfer bottleneck: Weeks 7–8 critical, some students struggle
Design challenges: Develop synthetic capacity, not just analysis	Experimental validation gap: *in silico* design without *in vivo* feedback

### 6.3 Limitations and extensions

COT has inherent limitations that define its proper domain while pointing toward productive extensions. Understanding these boundaries clarifies when COT is useful and when complementary approaches are needed.

COT’s qualitative abstraction from kinetics is the most significant limitation. COT identifies organizationally viable configurations but says nothing about reaction rates, concentrations, or temporal dynamics. For many design questions, qualitative answers suffice: knowing a metabolic pathway cannot form an organization (due to cofactor imbalance or lack of closure) indicates failure regardless of kinetics. However, real applications require kinetic details. The natural solution is sequential workflow: COT identifies structurally viable designs, eliminating those that cannot work regardless of parameters, then kinetic modeling optimizes viable designs.

A second limitation concerns stochasticity. COT deals with deterministic situtions (this or that process occurs), yet biological systems exhibit significant stochastic fluctuations (Elowitz and Leibler, 2000). These fluctuations can destabilize deterministically stable organizations or enable escape from organizational dynamics. Extending COT to stochastic networks requires defining organizational concepts probabilistically: what does self-maintenance mean when production and consumption are probabilistic? Preliminary approaches treat organizations as high-probability state space regions rather than invariant sets (Veloz et al., 2023).

The gap between organizational predictions and experimental validation remains pressing from an educational perspective. While mathematically rigorous, practical utility depends on whether organizational analysis predicts which designs succeed or fail. Currently, this rests on intuitive arguments rather than systematic validation. Needed are collaborative projects comparing organizational predictions with experimental outcomes: Does pathway X, predicted to lack organization, fail *in vivo*? Does consortium Y, predicted organizationally fragile, collapse under perturbation? Such studies would validate the framework and strengthen pedagogical impact.

From an educational perspective, our implementation has relied on qualitative observation and formative assessment rather than validated instruments for measuring changes in systems thinking. Future pedagogical research should include pre/post assessments using validated tools (adapting, for example, the Biology Systems Thinking framework of (Merrill et al., 2022) to organizational reasoning), comparisons with alternative approaches to teaching systems-level synthetic biology design, and longitudinal tracking of whether organizational thinking persists in students’ subsequent work. The development of a concept inventory for organizational reasoning in synthetic biology—analogous to existing biology concept inventories (Smith et al., 2008)—would enable rigorous cross-institutional comparison of pedagogical approaches.

## 7 Conclusion

Synthetic biology has evolved from assembling parts to engineering living systems. This transformation requires corresponding evolution in how we teach the field. Chemical Organization Theory provides the theoretical and pedagogical foundation this evolution demands, and can be extended to multiple other areas, especially interdisciplinary ones.

COT addresses fundamental challenges in synthetic biology education: It provides a very useful formal conceptual framework—the same principles apply from genetic circuits to synthetic ecosystems. It makes abstract concepts operational—self-organization, emergence, resilience become computable design criteria. It develops systems thinking—students learn to reason about circular causality, multi-level dynamics, and organizational viability.

Four years of implementation across diverse student populations demonstrates that COT-based curricula successfully develop the competencies modern synthetic biology requires. Students learn to think organizationally, enabling them to design systems that self-organize, predict which designs will fail before construction, and engineer resilience through organizational flexibility rather than rigid optimization.

As synthetic biology continues maturing, universal principles of organization and adaptation deserve a place in curricula alongside the essential molecular and genetic foundations. COT provides one formal framework for this purpose—identifying structurally viable designs as a necessary first step before kinetic optimization and experimental validation. Indeed, our pedagogical experience suggests that the interdisciplinary route—learning organizational reasoning in familiar domains before transferring to biology—may be among the most effective paths into synthetic biology’s complex adaptive systems challenges. The next-generation of synthetic biologists will not merely assemble parts—they will engineer organizations (Levin, 2023). We can help teach them how.

## Data Availability

Publicly available datasets were analyzed in this study. This data can be found here: https://pycot.utem.cl/.
